# Recurrence of histiocytic necrotizing lymphadenitis: A case report and literature review

**DOI:** 10.3892/etm.2014.1559

**Published:** 2014-02-19

**Authors:** LINTAO BI, JUN LI, ZHENXIA LU, YUMEI LIN, DAN WANG

**Affiliations:** Department of Hematology and Oncology, China-Japan Union Hospital Attached to Jilin University, Changchun, Jilin 130031, P.R. China

**Keywords:** recurrence, histiocytic necrotizing lymphadenitis

## Abstract

Histiocytic necrotizing lymphadenitis (HNL) is a unique form of self-limiting lymphadenitis with an unknown cause. The majority of cases resolve within several months and the disease has a low recurrence rate of 3–4%. In the present study, a prolonged recurrent case of HNL was reported. A 44-year-old female developed recurrent HNL with generalized lymphadenopathy 14 years after the original episode.

## Introduction

Histiocytic necrotizing lymphadenitis (HNL), also known as Kikuchi-Fujimoto disease, was first described in Japan in 1972 (1,2). HNL is a benign syndrome most commonly involving cervical lymphadenopathy, fever and night sweats. The etiology of HNL is unknown but it is hypothesized to be triggered by an autoimmune or viral process with an exaggerated T cell-mediated immune response (3–5). Definite diagnosis of HNL is made only via histopathological analysis from an open biopsy of the affected lymph nodes (6). The prognosis of HNL is generally favorable. The majority of patients with HNL have a self-limited course of the disease that resolves within several months, with a low recurrence rate of 3–4% (3). There is no specific treatment for HNL, but in severe cases, the use of corticosteroids has been recommended (7). The present study reports on a case of delayed recurrence of HNL with generalized lymphadenopathy 14 years after the original episode.

## Case report

A 30-year-old Chinese female presented with progressive fever and multiple lymph node enlargement (cervical, axillary and inguinal). The patient had daily fevers up to 103°F and presented with a sore throat, cough and fatigue. On physical examination, the patient exhibited significant and tender cervical, axillary and inguinal lymphadenopathy, with the largest node being 3×1.5 cm located at the left cervical region. The complete blood count revealed leukopenia, with a white blood cell count of 3,100 cells/mm^3^ and an increase in the lymphocyte ratio with 41% lymphocytes. Additional testing revealed elevated lactate dehydrogenase (LDH), β2 microglobulin (B2-MG) and erythrocyte sedimentation rate (ESR), while IgG was minimally elevated. Thyroid-stimulating hormone (TSH) was significantly decreased. The antinuclear antibody (ANA) test was negative, while C-reactive protein and liver function tests were normal, as shown in Table I. Infectious etiologies, including tuberculosis, human immunodeficiency virus (HIV), Epstein-Barr virus (EB virus), adenovirus, respiratory syncytial virus, influenza virus, parainfluenza virus, mycoplasma, cytomegalovirus, hepatitis A, hepatitis B and hepatitis C were negative.

**Figure 1:**
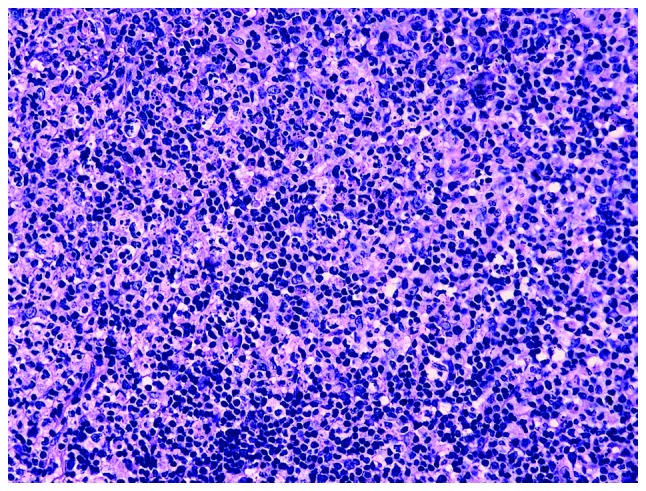
Cervical lymph node biopsy showed disrupted architecture, necrosis and nuclear debris (hematoxylin and eosin staining; magnification, ×200).

**Figure 2:**
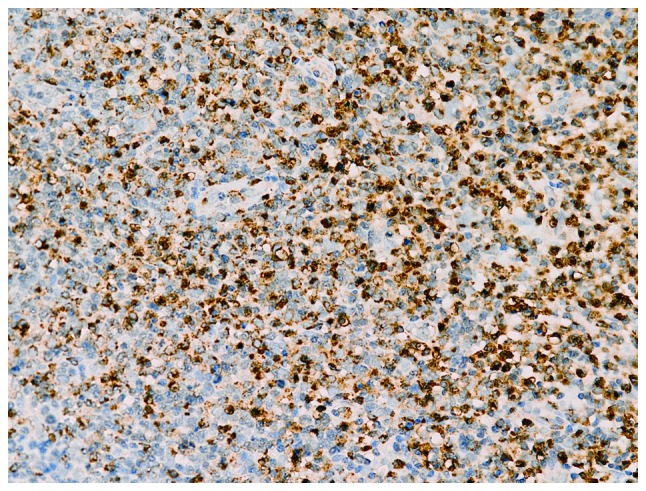
Immunohistochemistry was positive for CD68 (EnVision; magnification, ×200).

Ultrasound examinations revealed enlargement of multiple lymph nodes at the cervical, axillary and inguinal regions. In addition, ultrasound revealed homogeneously enhancing lesions, while the central hilar architecture of the lymph nodes had disappeared. There were hypoechoic areas on both thyroid lobes. Spiral lung computed tomography revealed multiple mediastinal lymph node enlargements; the largest was 11×11 mm. An excisional right cervical lymph node biopsy was performed and the results confirmed the diagnosis of HNL. Hematoxylin and eosin staining showed the lymph nodes were replaced by multifocal areas of necrosis and an abundance of cellular debris was present in the necrotic areas, as shown in Fig. 1. Immunohistochemical staining demonstrated the presence of CD3^+^, CD4^+^, CD8^+^, CD20^sparsely+^, CD21^+^, Ki-67^30%+^ and CD68^+^ (Fig. 2). Subsequently, the patient received treatment with dexamethasone (5 mg/day via i.v. drip). After one week of therapy, the body temperature returned to normal and rapid improvement was observed regarding lymphadenopathy. One month later, ESR, hemogram, IgG, TSH, LDH and B2-MG returned to normal levels and the enlarged lymph nodes regressed completely. The dose of dexamethasone was gradually reduced and stopped after 2 months.

A review of patient history revealed a diagnosis of HNL 14 years previously. During the first examination, the patient presented with high fever and cervical lymphadenopathy of one-week duration. An excisional cervical lymph node biopsy was performed and the results confirmed a diagnosis of HNL. The patient received prednisolone treatment, resulting in rapid remission in lymphadenopathy and fever. The patient was discharged with a full recovery.

## Discussion

HNL is a benign self-limiting condition that causes lymphadenopathy, most commonly observed in adults younger than 40 years of age. A female predominance has been reported (8). The majority of patients present with cervical lymphadenopathy and the next most common clinical manifestation is fever. Other less commonly reported observations include leukopenia, atypical lymphocytes on peripheral smear, liver dysfunction bone marrow involvement, fatigue, hepatosplenomegaly and skin rash (3,9,10). A definite diagnosis of HNL may be made reliably only via histopathological analysis from an open biopsy of the affected lymph nodes (7). There is no specific treatment for HNL, although in severe cases, the use of corticosteroids has been recommended to prevent a fatal outcome (7). Signs and symptoms associated with HNL usually resolve after several months (3,6,7).

A low recurrence rate of 3–4% has been reported (3,6,7) and recurrence has been recorded over a period of two to 12 years following initial presentation (11,12). The patient in the present study recurred 14 years after the initial onset, which represents the longest delayed recurring case of HNL. Patients with recurrent episodes were more likely to present with fever, cough and fatigue with frequent extranodal involvement at the initial presentation (13). Although the disease is characterized by regional lymphadenopathy, few patients show generalized lymphadenopathy. In the present case, there was simultaneous involvement of the cervical lymph nodes with the axillary and inguinal lymph nodes, even the mediastinal lymph nodes were involved at recurrence, indicating a generalized lymphoma that often leads to a misdiagnosis. In the present case, a diagnosis of HNL was confirmed according to the results from the pathological slices.

The etiology of HNL recurrence is unknown, but certain viral infections, including EB virus, parvovirus B19 or human herpes virus 8, have been hypothesized to be triggers for the relapse of HNL (14–16). Stéphan *et al* (17) observed that the recurrence of HNL was associated with the persistence of EB viral infection. Atarashi *et al* (18) reported a case of recurrent HNL in a human T lymphotropic virus type I carrier. For the present case, infectious etiologies, including EB virus, cytomegalovirus and HIV, were all negative. It is unknown whether other viral infections were associated with HNL in the present patient.

An association between recurrent HNL and autoimmune diseases has been reported. Cheng *et al* (19) described the clinical manifestations and outcomes of 195 patients diagnosed with HNL. A total of 14 of 96 patients (14.6%) had clinical recurrence of HNL, five of which developed an autoimmune disease, such as systemic lupus erythematosus (SLE). Individuals with HNL have been hypothesized to be more susceptible to SLE, thus, should be routinely screened for this disorder (3). HNL may precede, follow or coincide with the diagnosis of SLE. Londhey *et al* (20) reported a case that was initially diagnosed with HNL and SLE simultaneously. The patient was presently in remission following treatment for SLE. The fluorescence ANA test is useful in predicting patient prognosis. However, there is a possibility that recurrent disease with positive FANA may reflect the overlap between SLE and HNL (13). Lozano Parras *et al* (21) presented a case of HNL associated with subacute lymphocytic thyroiditis. In the present case, the patient showed a significant decrease in TSH levels and ultrasound revealed hypoechoic areas on both lobes of the thyroid, indicating possible concurrent thyroiditis.

In conclusion, the present study reported a case of HNL with a prolonged relapse of 14 years. The patient exhibited generalized lymphadenopathy, which included enlarged mediastinal lymph nodes. The patient responded well to a glucocorticoid regime and a full recovery was achieved in the initial and recurrent onsets.

## Figures and Tables

**Table I:** Laboratory data at the initial evaluation.

Variable	Admission value	Reference range
WBC (per mm^3^)	3,100	4,000–10,000
ESR (mm/h)	46	0–20
Lymphocytes (%)	41	20–40
CRP (mg/dl)	0.24	<0.80
ANA	Negative	Negative
B2-MG (mg/l)	2.75	0.91–2.2
LDH (IU/l)	251	91–180
TSH (mIU/l)	0.0005	2–10
IgG (g/l)	16.6	9.5–12.5
AST (IU/l)	32	0–40
ALT (IU/l)	27	0–40

WBC, white blood cell; ESR, erythrocyte sedimentation rate; CRP, C-reactive protein; ANA, antinuclear antibody; B2-MG, β2 microglobulin; LDH, lactate dehydrogenase; TSH, thyroid-stimulating hormone; AST, aspartate aminotransferase; ALT, alanine aminotransferase.
